# Plasma and complement proteins are essential for the antimicrobial activity of canine platelet lysate

**DOI:** 10.3389/fvets.2025.1605649

**Published:** 2025-07-01

**Authors:** Melikasadat Mollabashi, Alonza Klopfer, Thainá Lunardon, Nikolia Darzenta, Emily Davis, Matt Murray, Scarlett M. Sumner, Maria C. Naskou

**Affiliations:** ^1^Scott-Ritchey Research Center, College of Veterinary Medicine, Auburn University, Auburn, AL, United States; ^2^Department of Pathobiology, College of Veterinary Medicine, Auburn University, Auburn, AL, United States; ^3^Department of Clinical Sciences, College of Veterinary Medicine, Auburn University, Auburn, AL, United States

**Keywords:** canine platelet lysate, leukocyte concentration, complement proteins, plasma, antimicrobial properties

## Abstract

**Introduction:**

Platelet derived products have gained increasing attention as promising alternative biologicals for the treatment of canine wounds. Specifically, platelets play a crucial role during the inflammatory phase of wound healing due to the release of chemokines, proteins, cytokines, and growth factors. Additionally, platelets possess antimicrobial properties, which can be influenced by their manufacturing process, platelet and leukocyte concentration, activation method, and the presence of plasma and complement. The objective of this study was to assess how various preparation methods of platelet products affect their antimicrobial effect against bacteria commonly isolated from wounds.

**Methods:**

In this study, blood was collected from eight purpose-bred dogs, and platelet-rich plasma was produced using two methods of centrifugation, one leukocyte-enriching and one leukocyte-reducing. Some samples were processed for plasma depletion and platelet lysate was subsequently generated through freeze–thaw cycles. Additionally, portions of platelet lysate samples underwent heat treatment for complement inactivation. All treatment groups were tested against four common bacteria found in canine skin wounds: *Escherichia coli*, *Enterococcus faecalis*, *Staphylococcus pseudintermedius,* and *Staphylococcus aureus*. The antimicrobial effect of various lysate formulations was evaluated using a bacteria-spiking (time-killing) assay.

**Results:**

Platelet lysate significantly reduced the number of *S. aureus* and *E. coli* after 3 h compared to culture media. No significant differences were noted in the log reduction of bacteria between the centrifugation techniques. After depleting plasma, the log reduction of *S. pseudintermedius* was significantly less than before plasma depletion, whereas the opposite was seen for *E. faecalis* after 3 h. Complement-depleted plasma led to a significantly lower log reduction for *E. faecalis* after 24 h compared to platelet lysate.

**Discussion:**

Therefore, the presence of plasma and complement proteins in platelet lysate appear to play a critical role in inhibiting the growth of certain bacterial strains, whereas the leukocyte concentration does not have a significant effect. Further research is needed to identify the ideal formulation and dose of canine platelet lysate as an antimicrobial and wound healing treatment.

## Introduction

Wound healing is a complex multicellular dynamic event characterized by several processes such as inflammation, coagulation, epithelization, tissue regeneration, and granulation tissue formation (1). Platelets play a crucial role during the inflammatory phase of wound healing due to their alpha granules releasing most of the chemokines, proteins, cytokines, and growth factors responsible for activating and recruiting other cells involved in inflammatory responses (2). Platelet-rich plasma (PRP) is a platelet-derived product containing an increased concentration of platelets, widely known to benefit wound healing processes and inhibit common pathogens found in wounds (3–7). Platelet concentrates, such as PRP, can be subjected to several manufacturing techniques that include lysis of platelets to release their chemotactic and growth factors (8). The resulting product is called platelet lysate (PL) (8).

PL is an acellular product containing platelet-derived growth factors that can serve as an off-the-shelf alternative to PRP (9). As it is acellular, issues of immunogenicity are reduced, which is another attractive attribute of PL as a therapeutic (9). Additionally, PL can be pooled from several donors to decrease donor variability and can be stored for extended periods in the freezer (9–11). Another attractive aspect of PL regarding wound healing is its antimicrobial potential. Specifically, the antimicrobial properties originate from the release of antimicrobial peptides (AMPs) and an array of growth factors (12–15). The activity of AMPs has been shown to initiate pore formation within bacterial membranes, which can evoke cell death (16). New studies have demonstrated that canine platelet-derived products display antimicrobial properties in wounds infected with antibiotic-resistant Gram-negative (17) and *Methicillin-resistant Staphylococcus aureus* (*MRSA*) (18), while equine PL was recently found to inhibit the growth of *Enterococcus faecalis* and *Staphylococcus aureus* (8). Additionally, equine PL has shown both concentration-dependent and independent inhibition effects depending on the type of bacteria being treated (8). Therefore, PL has dual treatment potential, since it can be used for wound healing purposes, and as an alternative to traditional antibiotics for regulating wound-related bacterial infections, including those with antibiotic resistance.

PRP and PL can be manufactured in different ways, including various commercial systems, manual lab centrifugation methods, gravity filtration methods, and plateletpheresis (19). Final platelet concentrations of PRP vary greatly within the literature, even when using a single method of production (20, 21). Variability also exists regarding the leukocyte concentration (20, 21) and the method of platelet activation (22, 23). The variability of platelet products in the manufacturing processes and the inflammatory cytokine concentration, growth factors, and cellular composition can affect tissue metabolism *in vitro* and *in vivo* (7, 22–25). These factors can ultimately alter the efficacy of the final product.

Similarly, the antimicrobial potential of platelet products can be affected by the concentration of leukocytes, however, the results are controversial. Some studies showed that leukocytes were not essential for maintaining the antimicrobial properties of platelets (17, 26, 27). Specifically, leukocytes can produce pro-inflammatory protease and acid hydrolases that might induce an inflammatory response (28). On the contrary, some studies found that the presence of leukocytes in platelet products amplified their ability to inhibit bacterial growth (29, 30), because leukocytes produce myeloperoxidase that kills bacteria (31).

In additional to the platelet themselves, plasma can accelerate the wound healing process as well as provide antimicrobial properties (32). One specific component that likely contributes to its antimicrobial effect is the complement system. The complement system is comprised of unique plasma proteins that interact to opsonize pathogens while employing inflammatory responses that correspond to invading infections (33) and act as an extracellular and intracellular defense mechanism (34). Certain complement proteins, such as C3 and C5, can initiate cell differentiation, maintain immunological tolerance, and activate cell mediate responses (34). The literature demonstrates that specific plasma proteins, specifically C3a, contribute to the direct killing of *Escherichia coli*, *Pseudomonas aeruginosa,* and *Enterococcus faecalis* by binding and inducing breaks in bacterial membranes (35). Finally, Burnouf et al. found that complement activation pathways are essential for the antimicrobial function of human plasma and platelet-derived products against certain bacteria such as *Escherichia coli*, *Staphylococcus aureus*, *Pseudomonas aeruginosa,* and *Klebsiella pneumoniae* (36).

This study aimed to compare the antimicrobial activity of canine PL generated by different methods, including a leukocyte-reduced and a leukocyte-rich method, against bacteria commonly present in canine skin wounds. Subsequently, we aimed to identify whether the presence of plasma and complement are responsible for the antimicrobial action of PL. We hypothesized that canine PL would display antimicrobial effects against common bacteria found in canine skin wounds compared to standard bacteria growth media and that plasma and heat-sensitive components- namely the complement system- would improve its effectiveness.

## Materials and methods

### Canine blood donors

In this study, eight canine blood donors were recruited for whole blood donation. Dogs were purpose-bred beagles, four females and four males, with a mean age of 5.67 +/− 0.5 years and a mean weight of 14.83 +/− 2 kg. All donors were considered healthy based on history, preliminary blood work (complete blood cell count and chemistry panel), and physical examination. The study’s protocol (2022–5,085) was approved by the Auburn University Institutional Animal Care and Use Committee (IACUC).

### Generation of platelet concentrates

Approximately 100 mL of blood was collected in ACD-A tubes from each canine donor. A portion of the blood was also collected into an EDTA tube for a complete blood count (CBC) using the Heska hematology analyzer (Heska Element HT5, Heska, Colorado, United States). Platelet rich plasma (PRP), leukocyte-rich platelet rich plasma (L-PRP), and platelet-poor plasma (PPP) were manufactured under a biosafety laminar flow cabinet (Thermo Scientific Biological Safety Cabinets) (37). Briefly, two different manual centrifugation methods were used to isolate platelets from whole blood. For PRP, whole blood was centrifuged at 1000 g for 5 min. The plasma layer was then collected and centrifuged at 1500 g for 15 min to pellet the platelets. For the L-PRP, whole blood was centrifuged at 180 g for 20 min. The plasma layer and buffy coat were then collected and centrifuged at 650 g for 15 min to pellet the platelets. For each method, the PPP was removed without disturbing the platelet pellet, leaving two milliliters remaining for pellet resuspension. The platelet pellet was resuspended, and a platelet count was performed. All platelet concentrations were adjusted to 0.8–1 × 10^6^ platelets/μl using PPP. Samples were stored at −80°C.

### Plasma depletion

Before storage at −80°C, a portion of the PRP, L-PRP, and PPP samples were plasma depleted by centrifugation (Sorvall X Pro Series Centrifuge, ThermoFisher Scientific, Osterode am Harz, Germany) at 3000 g for 30 min. Plasma was removed without disturbing the pellet and the pellet was resuspended with an equal volume of phosphate-buffered saline without calcium and magnesium (PBS −/−, VWR, United States) to produce leukocyte-reduced platelet pellet concentrate (PPC) and leukocyte-rich platelet pellet concentrate (LPPC). Samples were then stored at −80°C.

### Generation of platelet lysates

PPP, PRP, LPRP, PPC, and LPPC formulations from eight dogs were thawed and pooled to generate three lots of each formulation, each containing lysate from three donors. All samples then underwent four additional freeze–thaw cycles using liquid nitrogen and a dry bath at 37°C (Precision GP 20, ThermoFisher Scientific, Newington, NH). Samples were placed in a microcentrifuge (Fresco 21 Centrifuge, ThermoFisher Scientific, Osterode am Harz, Germany) at 20,000 g for 20 min. The supernatant was collected as the final products: processed PPP (PPP), leukocyte-reduced platelet lysate (PL), leukocyte-rich platelet lysate (LPL), leukocyte-reduced platelet pellet lysate (PPL), and leukocyte-rich platelet pellet lysate (LPPL). Samples were stored at −80°C.

### Heat treatment (complement inactivation)

Heat treatment was performed on a portion of the PL, LPL, PPL, and LPPL formulations via a high-heat dry bath at 56°C for 30 min, followed by an immediate ice bath for 5 min (37). Samples were placed in a microcentrifuge at 10,000 g for 15 min at 4°C. The supernatant was collected to provide the final products: heat-treated leukocyte-reduced platelet lysate (hPL), heat-treated leukocyte-rich platelet lysate (hLPL), heat-treated leukocyte-reduced platelet pellet lysate (hPPL), and heat-treated leukocyte-rich platelet pellet lysate (hLPPL). Samples were stored at −80°C.

### Spiking assay (time-killing assay)

The spiking assay was performed with four clinically isolated bacteria obtained from ATCC: *Escherichia coli* (*E. coli;* ATCC #25922), *Enterococcus faecalis* (*E. faecalis*; ATCC #19433), *Staphylococcus pseudintermedius* (*S. pseudintermedius*; ATCC #49051), and *Staphylococcus aureus* (*S. aureus;* ATCC #12600). Experiments were performed under a biosafety hood using an aseptic technique. First, 1 mL of brain heart infusion broth (BHI; Becton Dickinson, Sparks MD) was used to rehydrate the freeze-dried bacteria. Resuspended bacteria were incubated at 37°C overnight with shaking at 200 rpm in a culture tube with a total of four milliliters of BHI broth. Following overnight incubation, the optical density (OD) was measured for each culture tube using duplicate aliquots in 96-well plates via Biotek Gen5 (Synergy HI Microplate Reader, Agilent Technologies Inc. Winooski, VT) at OD_600_. BHI broth was used as a positive control. Bacterial suspensions were adjusted to 0.3 OD, which corresponds to 10^8^ CFU/mL (38) prior to the spiking assay.

Each treatment group (PL, LPL, PPL, LPPL, hPL, hLPL, hPPL, hLPPL) at 90% v/v was added to a sterile tube containing the appropriate volume (10%) of adjusted bacterial stock suspension (10^8^ CFU/mL). BHI, PPP, and PBS were tested as positive and negative controls, accordingly. Treatments were incubated at 37°C under constant rotation (200 rpm) for 3 and 24 h. At each time point, cultures were serially diluted in PBS (−/−) (six 10-fold dilutions). Nutrient agar plates were inoculated with 200 μL aliquots from each dilution and incubated overnight at 37°C. Bacterial colonies were manually counted using a colony counter (Reichert Quebec Darkfield). Plates containing approximately 30–300 CFUs were selected for analysis from each experimental cohort.

### Statistical analysis

To reduce individual variability, platelet lysates from three dogs were pooled per lot and a total of three lots were generated (11). A power analysis showed that a sample size of 3 would be sufficient to test the antimicrobial properties of lysates to achieve a statistical significance of *p* < 0.05 and power = 90%. Assessments between spiking assay results were based on the log reductions compared to bacteria cultures in BHI [Log reduction = Log (bacterial count of positive control/bacterial count of treatment)]. All data were imported into a statistical analysis program (Graphpad Prism; Graphpad Software Inc. San Diego, CA, United States). Normality was assessed by the visual examination of histograms of the residual, normal plots of residuals, and via utilizing the Shapiro-Wilks test. The equality of variances was calculated utilizing Levene’s test and plotting residuals against the fitted value. Hypotheses were tested by repeated measures or 2-way analysis of variance (ANOVA). Tukey’s test was used to adjust for multiple paired comparisons. All statistical analysis was done at *p* < 0.05 level of significance. Continuous data were summarized and reported as mean ± standard deviation. Samples were analyzed in duplicates.

## Results

### Hematologic values

The mean concentration of platelets, white blood cells (WBC), and hematocrit (HCT), from the whole blood and platelet products are shown in Table 1. The mean (± SD) platelet concentration for whole blood was 239.375 ± 80.627 × 10^3^/μL, the WBC concentration was 7.89 ± 1.64 × 10^3^/μL, and the HCT was 53.32% ± 6.79. Compared to whole blood, there was an 11.8-fold increase in platelet concentration for PRP (2,837 ± 1335.605 × 10^3^/μL) and a 9.46-fold increase for L-PRP (2,266.6 ± 1494.311 × 10^3^/μL). The WBC concentration of PRP decreased 4.55-fold compared to whole blood (1.728 ± 6.69 × 10^3^/μL) while L-PRP had a 2-fold decrease in WBC concentration compared to whole blood (3.926 ± 2.842 × 10^3^/μL). There was minimal blood hemodilution for both PRP and L-PRP. The concentration of platelets, WBC, and HCT were negligible in all lysate formulation groups (PL, LPL, PPL, and LPPL; data not shown).

**Table 1:** Descriptive results of hematological data presented as mean ± standard deviation from whole blood, and platelet-derived concentrates.

Hematology analysis			
Preparation	PLT (x10^3^/μL)	WBC (x10^3^/μL)	HTC (%)
Whole blood	239.375 ± 80.627	7.871 ± 1.644	53.312 ± 6.79
Pure platelet concentrates			
PC	2,837 ± 1335.605	1.728 ± 6.69	0
Leukocyte-rich platelet concentrates			
LPC	2,266.6 ± 1494.311	3.926 ± 2.842	0

PLT, platelet concentration; WBC, white blood cell concentration; HTC, hematocrit; PRP, leukocyte-reduced platelet-rich plasma; L-PRP, leukocyte-rich platelet-rich plasma.

### Spiking assay

#### The effect of platelet concentration

**Figure 1:**
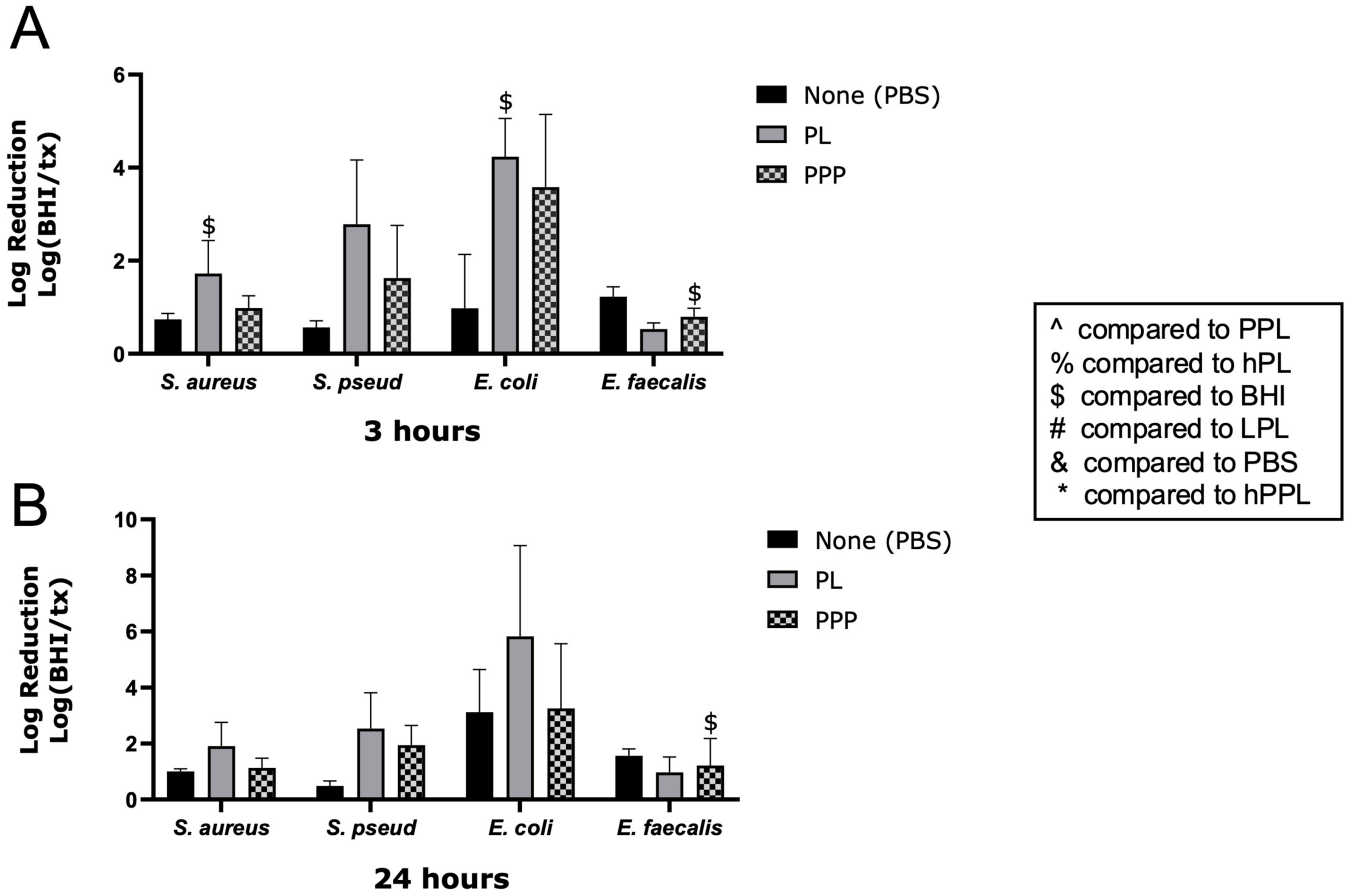
Log reduction of *S. aureus*, *S. pseudintermedius*, *E. coli*, and *E. faecalis* following the addition of PBS, PL, and PPP after 3 h (A) and 24 h (B). Data are presented as mean bacteria log reduction ± S. D. normalized to bacteria grown in BHI according to the following formula: Log (BHI/treatment). ^$^ *p* < 0.05 compared to BHI; *n* = 3 lots of pooled platelet lysate. PBS, Phosphate Buffered Saline; PL, Platelet Lysate; PPP, Platelet-Poor Plasma; PPL, leukocyte-reduced platelet pellet lysate; hPL, Heat-treated Platelet Lysate; BHI, brain heart infusion broth; LPL, Leukocyte-Rich Platelet Lysate; hPPL, Heat-treated leukocyte-reduced platelet pellet lysate.

The log reduction in growth of the *E. coli, E. faecalis*, *S. pseudintermedius*, and *S. aureus* after 3 and 24 h of PL or PPP treatment is demonstrated in Figure 1. PL caused a significant log reduction of *S. aureus* (1.724 ± 0.7120, *p* = 0027) and *E. coli* (4.238 ± 0.8227, *p* = 0.0486) after 3 h. However, this effect was not significant at 24 h. PPP resulted in a significant log reduction of *E. faecalis* after 3 (0.7937 ± 0.1841, *p* = 0.0220) and 24 h (1.215 ± 0.9646, *p* = 0.0149). A nonsignificant trend was noted for PL to achieve a greater log reduction than PPP for *E. coli, S. pseudintermedius*, and *S. aureus* after both 3 and 24 h. These findings suggest that platelets are essential for the antimicrobial action of PL against all bacteria except *E. faecalis*.

#### The effect of leukocyte concentration

**Figure 2:**
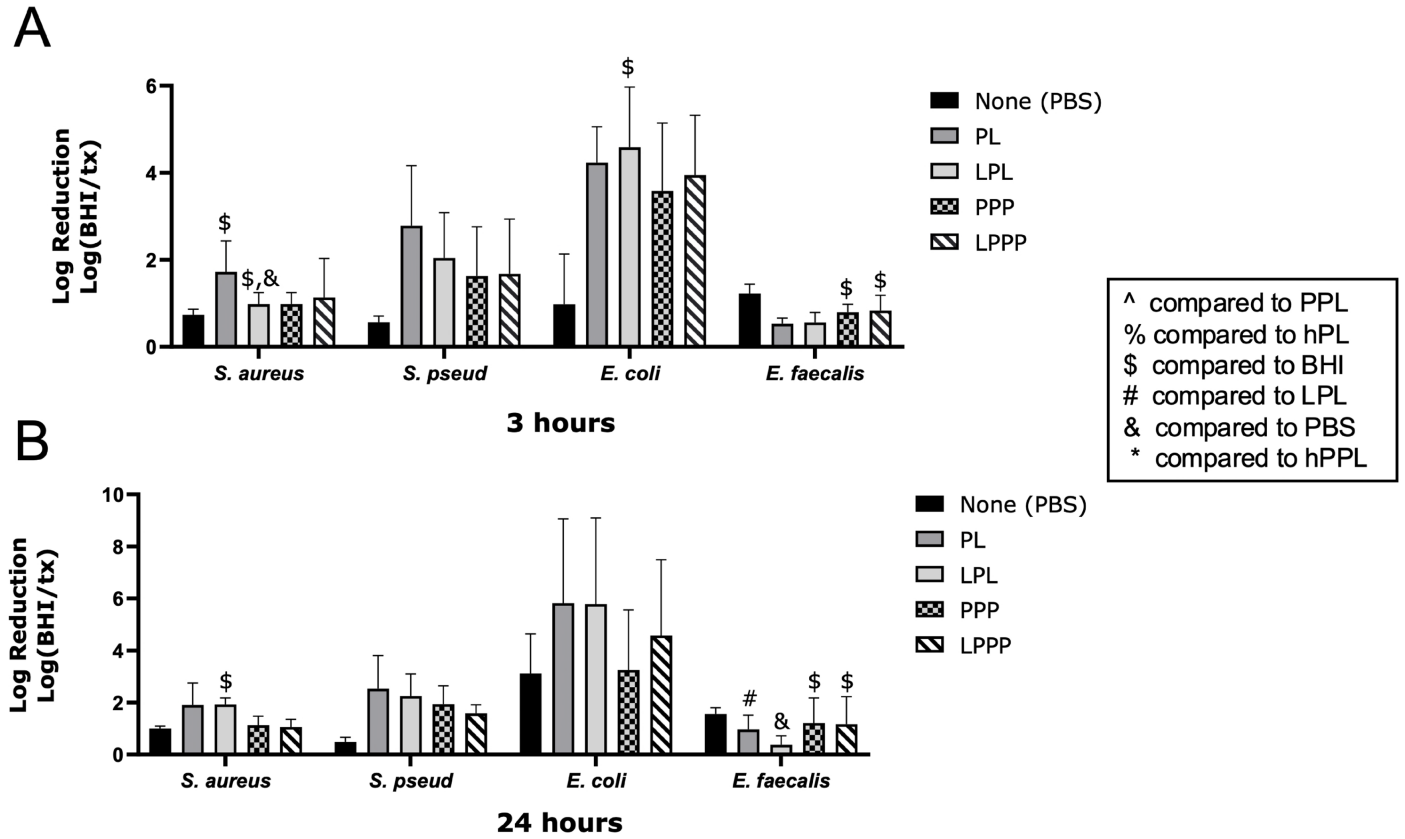
Log reduction of *S. aureus*, *S. pseudintermedius*, *E. coli*, and *E. faecalis* following the addition of PBS, PL, LPL, PPP, and LPPP after 3 h **(A)** and 24 h **(B)**. Data are presented as mean bacteria log reduction ± S. D. normalized to bacteria grown in BHI according to the following formula: Log (BHI/treatment). ^$^*p* < 0.05 compared to BHI; ^&^*p* < 0.05 compared to PBS; ^#^*p* < 0.05 compared to LPL; *n* = 3 lots of pooled platelet lysate generated from 8 donors. PBS, Phosphate Buffered Saline; PL, Platelet Lysate; LPL, Leukocyte-Rich Platelet Lysate; PPP, Platelet-Poor Plasma; LPPP, Leukocyte-Rich Platelet-Poor Plasma; PPL, leukocyte-reduced platelet pellet lysate; hPL, Heat-treated Platelet Lysate; BHI, brain heart infusion broth; hPPL, Heat-treated leukocyte-reduced platelet pellet lysate.

The log reduction of bacteria treated with PL and LPL is shown in Figures 2. LPL caused a significant log reduction of *S. aureus* at 3 (1.576 ± 0.1900, *p* = 0.0237) and 24 h (1.932 ± 0.2428, *p* = 0.0258). At 3 h, LPL had a significantly greater log reduction of *S. aureus* than PBS (*p* = 0.0120). LPL (0.3728 ± 0.3514) caused a significant, weaker, log reduction of *E. faecalis* compared to PL (0.9720 ± 0.5482, *p* = 0.0045) and PBS (1.561 ± 0.2435, *p* = 0.0174) after 24 h. LPPP (0.8369 ± 0.3485, 1.171 ± 1.058) and PPP (0.7937 ± 0.1841, 1.215 ± 0.9646) had a significant log reduction of *E. faecalis* at 3 (*p* = 0.0140, *p* = 0.0220) and 24 (*p* = 0.0192, *p* = 0.0149) hours. PL treatment exhibited a nonsignificant trend of a greater log reduction of *S. aureus* and *S. pseudintermedius* than LPL after 3 h, and for *S. pseudintermedius* after 24 h. The opposite trend, with LPL achieving a greater log bacteria reduction than PL, was noted for *E. coli* after 3 h. Therefore, leukocytes do not appear to affect the ability of platelets to inhibit bacterial growth.

#### The effect of plasma content

**Figure 3:**
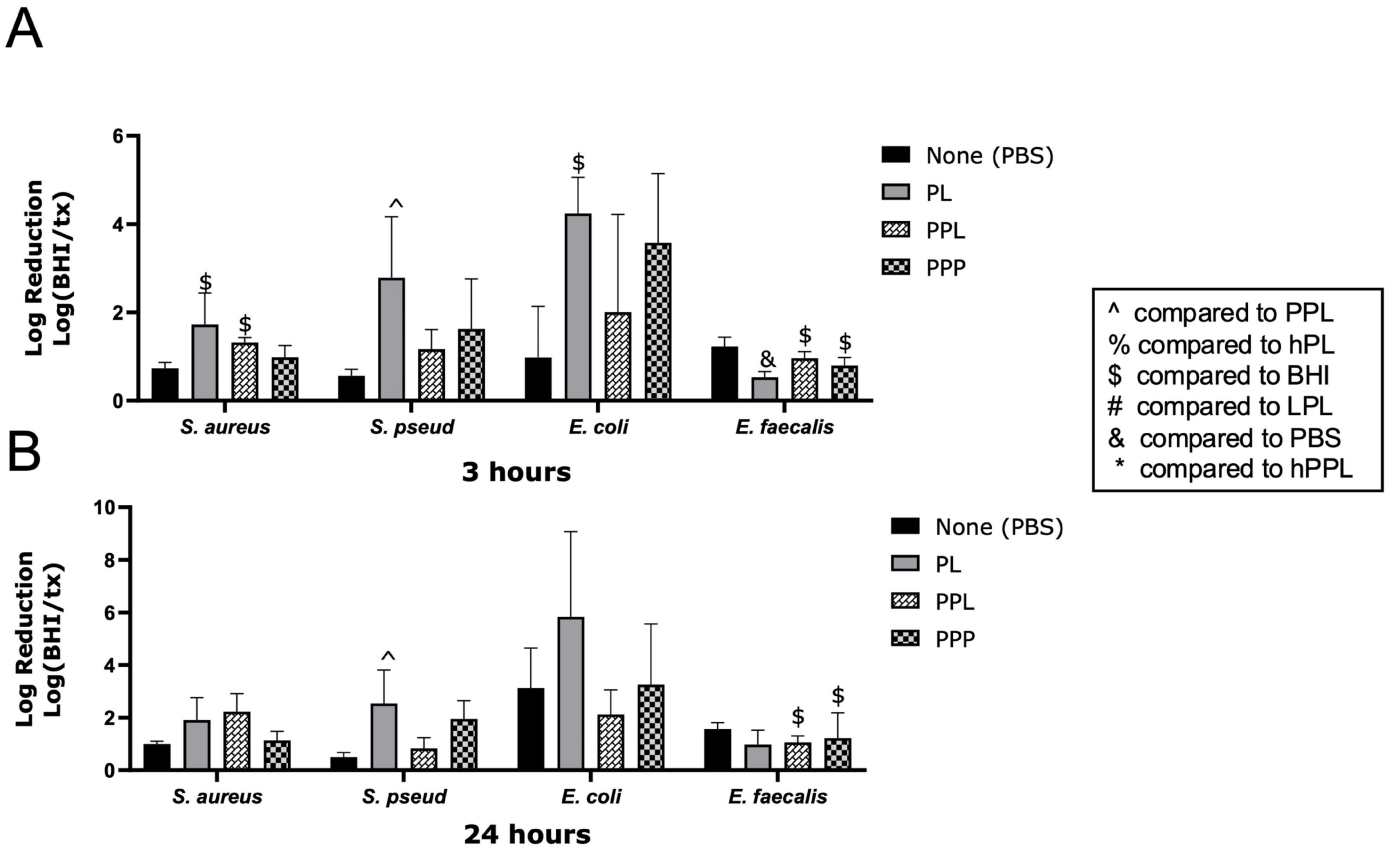
Log reduction of *S. aureus*, *S. pseudintermedius*, *E. coli*, and *E. faecalis* following the addition of PBS, PL, PPL, and PPP after 3 h **(A)** and 24 h **(B)**. Data are presented as mean bacteria log reduction ± S. D. normalized to bacteria grown in BHI according to the following formula: Log (BHI/treatment). ^$^*p* < 0.05 compared to BHI; ^*p* < 0.05 compared to PPL; *n* = 3 lots of pooled platelet lysate generated from 8 donors. PBS, Phosphate Buffered Saline; PL, Platelet Lysate; PPL, leukocyte-reduced platelet pellet lysate; PPP, Platelet-Poor Plasma; hPL, Heat-treated Platelet Lysate; BHI, brain heart infusion broth; LPL, Leukocyte-Rich Platelet Lysate; hPPL, Heat-treated leukocyte-reduced platelet pellet lysate.

The log reductions of bacteria in the presence of PL and PPL are illustrated in Figure 3. PPL caused a significant log reduction of *E. faecalis* at 3 (0.9623 ± 0.1493, *p* = 0.0396) and 24 h (1.057 ± 0.2413, *p* = 0.0372). At 3 h, the log reduction of *E. faecalis* was significantly greater with PBS than with PL (0.5296 ± 0.1331, *p* = 0.0260). In contrast, PL caused a greater log reduction of *S. pseudintermedius* at 3 (2.786 ± 1.377) and 24 h (2.538 ± 1,275) compared to PPL (1.166 ± 0.4440, *p* = 0.0478 and 0.8274 ± 0.4030, *p* = 0.0325). A nonsignificant trend toward greater log reduction with PL compared to PPL was noted with *S. aureus* and *E. coli* at 3 h and *E. coli* at 24 h (Figures 3A,B). These data demonstrate that plasma contributes to the antimicrobial properties of platelets.

#### The effect of complement inactivation

**Figure 4:**
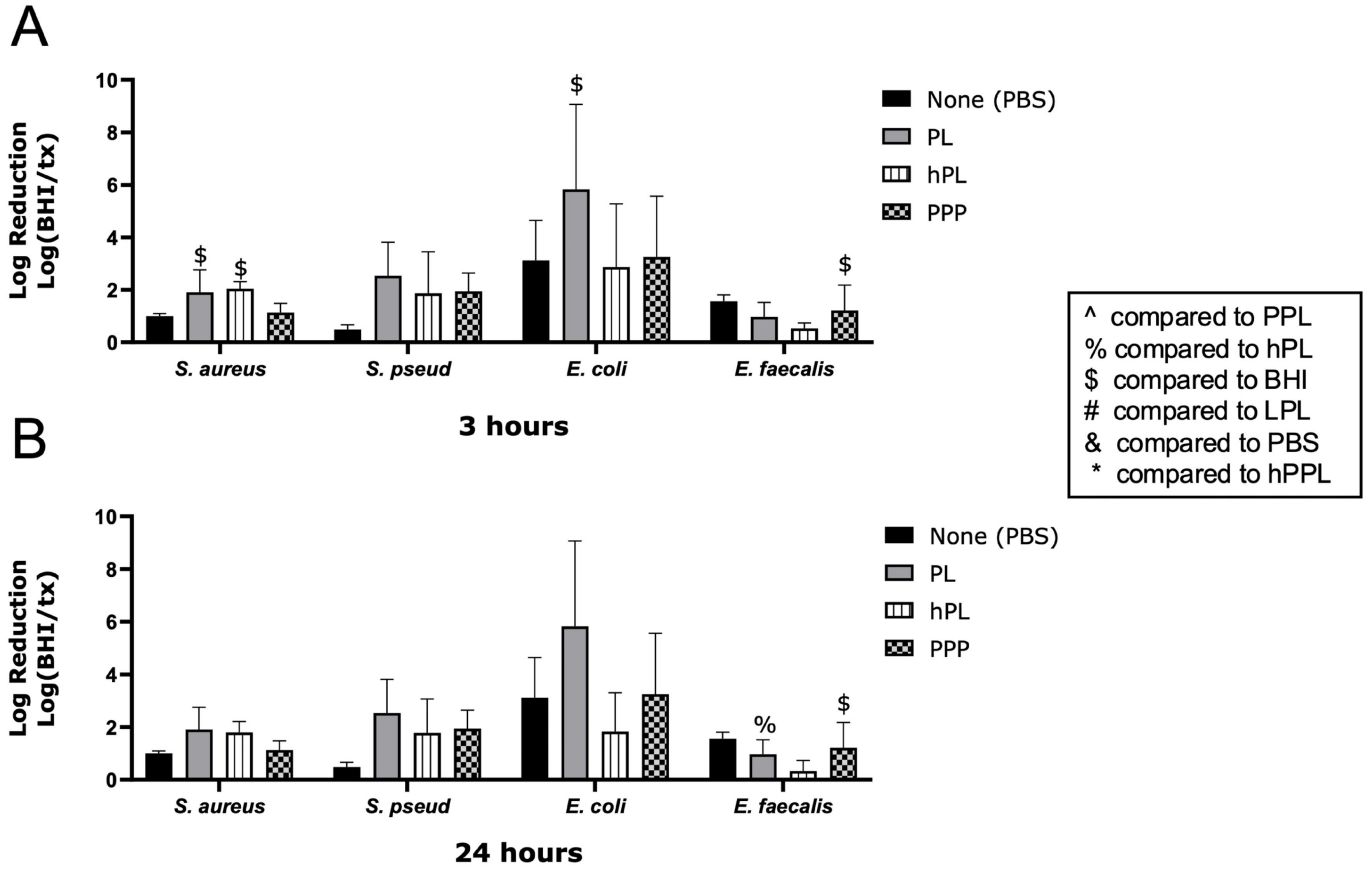
Log reduction of *S. aureus*, *S. pseudintermedius*, *E. coli*, and *E. faecalis* following the addition of PBS, PL, hPL, and PPP after 3 h **(A)** and 24 h **(B)**. Data are presented as mean bacteria log reduction ± S. D. normalized to bacteria grown in BHI according to the following formula: Log (BHI/treatment). ^$^*p* < 0.05 compared to BHI; ^%^*p* < 0.05 compared to hPL; *n* = 3 lots of pooled platelet lysate generated from 8 donors. PBS, Phosphate Buffered Saline; PL, Platelet Lysate; hPL, Heat-treated Platelet Lysate; PPP, Platelet-Poor Plasma; PPL, leukocyte-reduced platelet pellet lysate; BHI, brain heart infusion broth; LPL, Leukocyte-Rich Platelet Lysate; hPPL, Heat-treated leukocyte-reduced platelet pellet lysate.

The log reduction of bacteria with PL and hPL to compare the effects of heat treatment is depicted in Figure 4. At 3 h, hPL had a significant log reduction of *S. aureus* (2.049 ± 0.2612, *p* = 0.0267), which was significantly greater than the effect of PBS (0.7370 ± 0.1307, *p* = 0.0320). PL led to a greater log reduction of *E. faecalis* (0.9720 ± 0.5482) compared to hPL (0.3381 ± 0.3916, *p* = 0.0026) after 24 h. No significant differences were noted between PL and hPL at either time point for *E. coli* or *S. pseudintermedius* (Figures 3A,B). This data suggests that heat-sensitive plasma proteins are necessary for the antimicrobial features of platelets.

#### The effect of complement inactivation combined with plasma depletion

**Figure 5:**
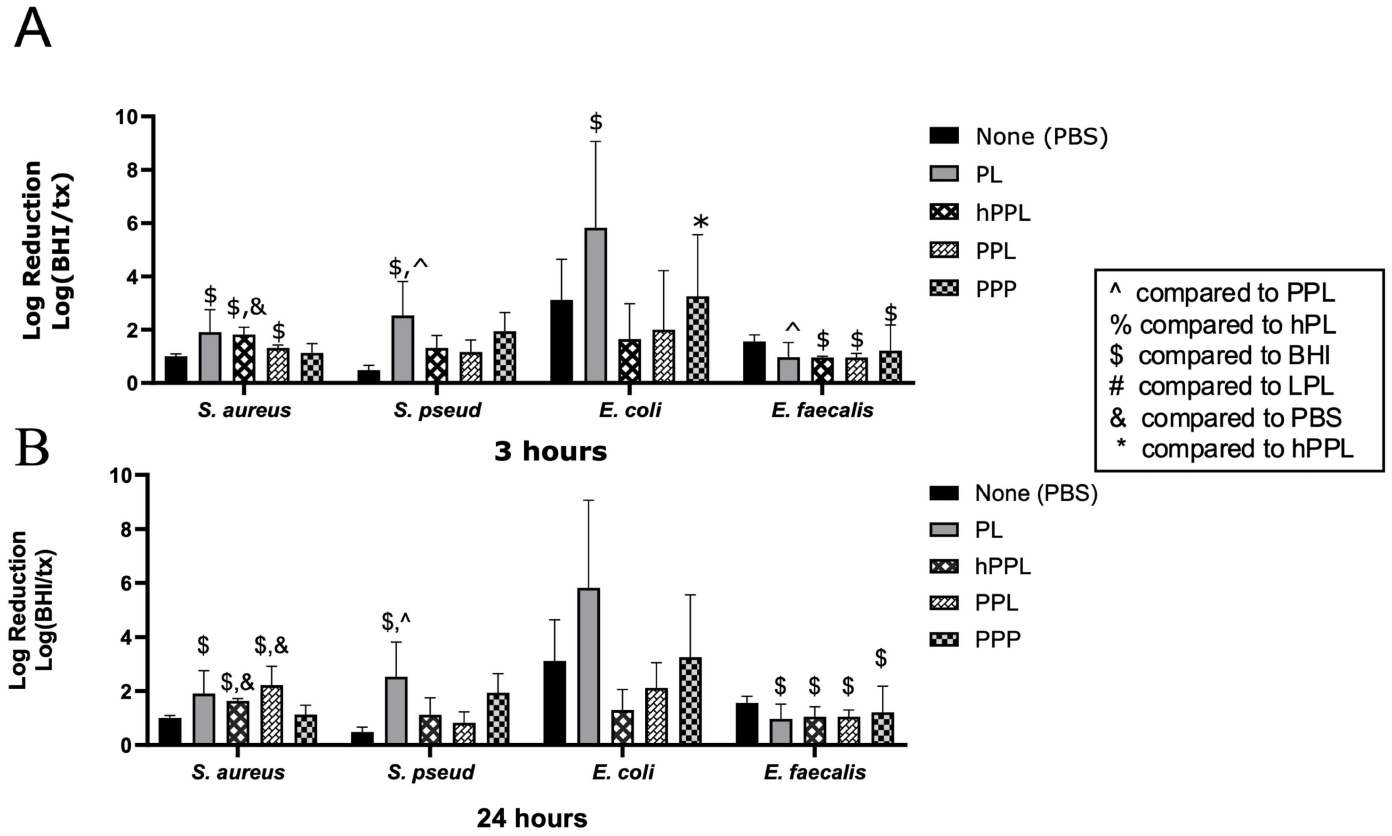
Log reduction of *S. aureus*, *S. pseudintermedius*, *E. coli*, and *E. faecalis* following the addition of PBS, PL, hPPL, PPL, and PPP after 3 h **(A)** and 24 h **(B)**. Data are presented as mean bacteria log reduction ± S. D. normalized to bacteria grown in BHI according to the following formula: Log (BHI/treatment). ^$^*p* < 0.05 compared to BHI; ^&^*p* < 0.05 compared to PBS; ^*p* < 0.05 compared to PPL; *p < 0.05 compared to hPPL; *n* = 3 lots of pooled platelet lysate generated from 8 donors. PBS, Phosphate Buffered Saline; PL, Platelet Lysate; hPPL, Heat-treated leukocyte-reduced platelet pellet lysate; PPL, leukocyte-reduced platelet pellet lysate; PPP, Platelet-Poor Plasma; hPL, Heat-treated Platelet Lysate; BHI, brain heart infusion broth; LPL, Leukocyte-Rich Platelet Lysate.

The log reduction of bacteria with PL and hPPL to compare the effects of plasma depletion combined with heat treatment is shown in Figure 5. Treatment with hPPL led to a significant log reduction of *S. aureus* at 3 h (1.819 ± 0.2719, *p* = 0.0368), which was also significantly greater reduction than PBS (0.7370 ± 0.1307, *p* = 0.0291), and at 24 h (; 1.001 ± 0.09561, *p* = 0.0049). Treatment with hPPL also led to a significant log reduction *E. faecalis* after 3 (0.9620 ± 0.04092, *p* = 0.0012) and 24 h (1.053 ± 0.3638, *p* = 0.0039). Conversely, the log reduction of *E. coli* after treatment with hPPL (1.645 ± 1.327) was significantly weaker compared to PPP (3.579 ± 1.566, *p* = 0.0282) after 3 h. PL was not significantly different from hPPL at 3 and 24 h, though trends were noted for PL to provide a greater log reduction of *E. coli* and *S. pseudintermedius.* Thus, the presence of plasma and its complement proteins is essential for maintaining the antimicrobial properties of platelets.

#### The effect of nutrient depletion

In order to evaluate whether the inhibition of the bacteria growth following the addition of platelet derived products could be partially due to nutrient deprivation we set up a serial of experiments evaluating bacteria growth in the presence of PL, PPP and PBS. Specifically, Bacterial colony counts for *S. aureus, S. pseudintermedius, E. coli and E. faecalis* after 3 and 24 h of PL, PPP, and PBS treatment are demonstrated in Supplementary Figure 1. We found that, PL led to a nonsignificant inhibition of both *S. aureus* (Supplementary Figure 1A) and *S. pseudintermedius* (Supplementary Figure 1B) compared to baseline. However, PPP inhibited growth of *S. pseudintermedius* but supported the growth of *S. aureus* compared to baseline after 3 and 24 h. Moreover, we found that the addition of PL, PPP, and PBS significantly inhibited the growth of *E. coli* after 3 h compared to the baseline. Similarly, after 24 h, PL, PPP, and PBS significantly decreased *E. coli* growth. PBS significantly supported the growth of *S. pseudintermedius* at 24 h. The same nonsignificant trend was noted for *S. aureus* after the addition of PBS for two time points. Finally, PL significantly and PPP non significantly increased the growth of *E. faecalis* compared to baseline after 3 h. The same trends were observed for PL and PPP after 24 h. Even though PBS decreased its growth compered to baseline after 24 h. Given that bacteria growth was encountered following the addition of PBS, it is not reasonable to believe that inhibition of bacteria growth is due to the lack of nutrients that are essential for bacteria growth.

## Discussion

In this study, highly concentrated PRP was achieved in both the leukocyte-reduced and leukocyte-rich formulations. The recommended platelet concentration for platelet concentrates is at least 300 × 10^3^ platelets/ μL for a therapeutic effect to occur based on the recommendation by the American Red Cross (22, 39). In this study, the number of concentrated platelets was consistent and exceeded the guidelines of the American Red Cross and previous studies (39). Due to the high concentration of platelets and standardization, the final concentration of all platelet products was standardized to 800–1,000 × 10^3^/μL prior to further processing. Additionally, leukocyte reduction in the leukocyte-reduced PRP was more successful compared to our previous work (37), which is in line with previous work by Shin et al. (22). However, leukocyte concentration was less successful in this study compared to previous studies using the leukocyte-rich method (17, 37). These variations are likely due to collection variations, donor variability, and processing time, particularly following the first centrifugation cycle.

When comparing PL and PPP, PL significantly reduced the growth of *S. aureus and E. coli*, and a trend was noted for PL to achieve a greater log reduction than PPP for *E. coli*, *S. pseudintermedius*, and *S. aureus.* These findings suggest that platelets are essential for the antimicrobial action of PL against *E. coli*, *S. pseudintermedius*, and *S. aureus*. Similar to PL, LPL demonstrated antimicrobial activity for *S. aureus*, *E. coli,* and *S. pseudintermedius* with a greater significance noted against *S. aureus* and *E. coli* at the 3-h timepoint. Significant antimicrobial activity against *E. faecalis* was not identified with treatment of PL or LPL. Our findings are consistent with the previous findings reported in the literature, noting platelet preparations inhibited *S. aureus* and *E. coli* (36), but not *E. faecalis* (40, 41). These results are also consistent with a previous study which proved leukocyte-reduced and leukocyte-rich platelet pellet concentrate have similar antimicrobial properties against five strains of bacteria (26). However, not all studies agree with this finding. Another study found the addition of neutrophils enhanced the antibacterial properties of platelet-rich plasma (30). Giusti et al. noticed that different leukocyte concentrations might have beneficial or detrimental impacts on the healing process, depending on the specific clinical circumstance (42). It is also worth noting that our results may be affected by the relatively low differential in leukocyte count between our formulations. Future studies should optimize the methodology used for leukocyte depletion during the manufacturing of platelet concentrates and guidelines may be needed for leukocyte concentrations depending on desired clinical applications.

Plasma has previously been noted to play a major role in the antibacterial properties of various orthobiologics (43). According to previous studies, the bactericidal properties of platelet products are mostly due to plasma factors (36). Other studies have also proved that the antimicrobial action of platelet products is related to the presence of plasma rather than platelets themselves (17, 36). Our results displayed that the depletion of plasma from PL (PPL) led to significantly less bacteria growth inhibition than PL after 3 and 24 h for *S. pseudintermedius* and *E. coli*. In contrast, PPL was more effective than PL against *E. faecalis* after 3 and 24 h and against *S. aureus* after 24 h. This latter finding is not in accordance with the previous literature, which concluded the antimicrobial properties of PRP against *E. faecalis* are due to the synergistic effects of plasma and platelet-derived factors (44). Additionally, in this study, plasma depletion inhibited the growth of *S. aureus* after 24 h. This might be due to the fact that *S. aureus* has a coagulase enzyme that converts fibrinogen to fibrin, which is used as an extracellular matrix to protect itself (45). By removing plasma, the above protection mechanisms are not available, which makes *S. aureus* more susceptible to therapeutic invasions. Thus, we believe that plasma is necessary for the antimicrobial properties of PL. Plasma contains protease, which seems critical for the cleavage of proteins in platelets. It is worth mentioning that the amount of plasma is a factor that needs to be considered carefully. In our study, platelet concentrates were resuspended in 100% plasma. However other studies found that variable plasma concentrations can affect the antimicrobial properties of the product (15, 46). Specifically, one study found that 10% of plasma is the most efficient concentration for maintaining an adequate amount of protease (15). Furthermore, another study demonstrated that 10% plasma is efficient and higher than 50% plasma is detrimental to the antimicrobial properties of platelets (46). Future studies should evaluate meticulously how various plasma concentrations can affect the antimicrobial features of platelet-derived products.

Based on previous studies, the heat-sensitive complement proteins within plasma are critical in inhibiting bacterial growth (27, 36). Our results further support this finding as heat-treated PL (hPL) did not provide the same bacterial growth reduction in any bacteria tested compared to PL. Accordingly, the presence of the protein complements seems crucial for maintaining the antimicrobial activity of PL. (47) Specifically, complement proteins use opsonin as labels on the bacteria to facilitate phagocytosis; in addition, they increase bacterial opsonization by antibodies since the antibodies kill the bacteria (33). Thus, our findings confirm the necessity of heat-sensitive complement proteins for maintaining the antimicrobial features of PL.

Combining plasma depletion and heat treatment was also evaluated. When plasma was depleted from PL, we found that plasma-depleted PL (PPL) was not efficient in inhibiting bacterial growth except for *E. faecalis*. Similarly, inactivating complements by utilizing heat had the same effect on PL (hPL). When we performed plasma depletion and heat treatment simultaneously, we confirmed a decreased potential of that formulation in inhibiting bacterial growth. Future studies on platelet products should consider the effects of plasma proteins and processing, including heat exposure, as these may change the product’s therapeutic potential.

To evaluate whether a nutritional deprivation is responsible for the increased bacteria reduction in the presence of platelet derived products we evaluated the growth of a specific concentration of bacteria in the presence of PL, PPP and PBS. We assumed that PBS would provide minimal notional support. According to our results, we confirmed that PL has a bactericidal effect against *E. coli* after both 3 and 24 h since it decreased the colony-forming units of a certain bacteria concentration more than 3 log 10 CFU/mL (28). Our finding is aligned with Burnouf et al. since a similar bactericidal activity of platelet preparations against *E. coli* was observed (36). In addition, both PL and LPL showed bacteriostatic activity against *S. aureus*, and *S. pseud* which is also consistent with previous studies (28). Thus, the antimicrobial effect of PL cannot be contributed to a nutrient deprivation effect.

Limitations of this study include a low differential between leukocyte concentrations in our “leukocyte-reduced” and “leukocyte-rich” preparation. This was likely affected by our inability to solely collect plasma from the top layer of the buffy coats, as contamination of leukocytes from accidentally aspirating the buffy coat is suspected. Protocol adjustments or use of different techniques or equipment may aid in obtaining the desired leukocyte concentration. Additional limitations include a small sample size, limited time points, and using commercially available bacterial strains collected from a human healthcare setting. In this study we decided to pool lysates generated from eight donors in an effort to decreased individual variability as has been previously reported (11).

The preparation process of platelet lysates included four freeze–thaw cycles in liquid nitrogen. It is possible that the number of freeze/thaw cycles could affect the release of factors from platelet granules and subsequently their biological activity. However, various studies have demonstrated that performing 3–5 freeze/thaw cycles achieves the optimal concentration of growth factors from platelet granules (48). Regardless, future studies should evaluate the effect of various numbers and conditions of freeze/thaw cycles for the optimal release of growth factors and antimicrobial properties. Finally, the generation process of lysates included the addition of acid-citrate-dextrose solution A (ACD-A) as an anticoagulant. Even though the addition of dextrose could have an impact on the composition of the growth factors, ACD-A is a common anticoagulant used in transfusion medicine for the preparation of platelet derived biologicals (49, 50). Specifically, a study evaluated the effect of various anticoagulants on the composition of growth factors and found that no differences were detected on PDGF concentration among the groups (49). However, a direct comparison has not been performed between ACD-A and acid-citrate to evaluate the effect of dextrose on factor composition.

Our results demonstrated that PL and LPL have antimicrobial properties independent of leukocyte concentration, specifically against *S. aureus*, *E. coli*, and *S. pseudintermedius*, but not *E. faecalis*. Plasma proteins and other plasma components seem to be critical for inhibiting the growth of some bacterial strains. In conclusion, platelet derived products have the potential to accelerate the wound healing process via the release of growth factors, anti-inflammatory and antimicrobial molecules. Future studies should investigate the most appropriate concentration of PL for targeted antimicrobial therapy and use in wound healing in dogs.

## Data Availability

The raw data supporting the conclusions of this article will be made available by the authors, without undue reservation.

## Supplementary material

Supplementary Figure 1 Mean number of bacterial colonies following treatment with PL, PPP, and PBS after 3 and 24 hours for *S. aureus* **(A)**, *S. pseudintermedius* **(B)**, *E. coli* **(C)**, and *E. faecalis* **(D)**. Data are presented as mean Colony Forming Unit per milliliter. The initial inoculum population was 108 CFUs/mL, represented by the dotted line (* = *p* < 0.05). *n* = 3 lots of pooled platelet lysate generated from 8 donors. PL, Platelet Lysate; PPP, Platelet-Poor Plasma; PBS, Phosphate Buffered Saline.
