# Supplementary figures and images for: Plasma and complement proteins are essential for the antimicrobial activity of canine platelet lysate
Source: Front Vet Sci. 2025 Jul 1;12:1605649. doi: 10.3389/fvets.2025.1605649 (PMC12259428; doi:10.3389/fvets.2025.1605649)

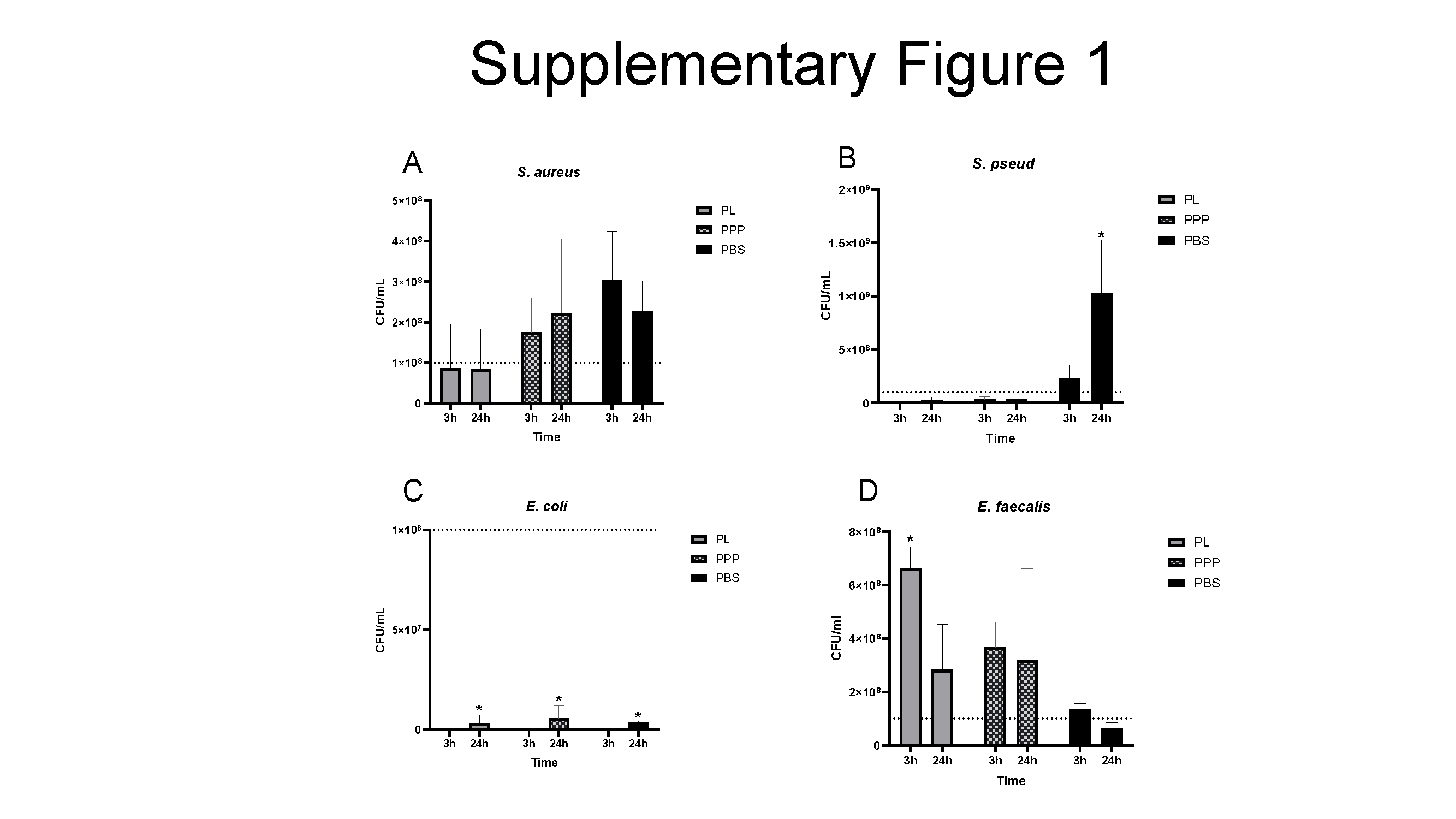

Supplement: Supplementary Figure 1 — Mean number of bacterial colonies following treatment with PL, PPP, and PBS after 3 and 24 hours for S. aureus (A), S. pseudintermedius (B), E. coli (C), and E. faecalis (D). Data are presented as mean Colony Forming Unit per milliliter. The initial inoculum population was 108 CFUs/mL, represented by the dotted line (* = p < 0.05). n = 3 lots of pooled platelet lysate generated from 8 donors. PL, Platelet Lysate; PPP, Platelet-Poor Plasma; PBS, Phosphate Buffered Saline. [file Image_1.TIFF]
